# Nutrigenomics and immune function in fish: new insights from omics technologies

**DOI:** 10.1016/j.dci.2017.02.024

**Published:** 2017-10

**Authors:** Samuel A.M. Martin, Elżbieta Król

**Affiliations:** Institute of Biological and Environmental Sciences, University of Aberdeen, Aberdeen AB24 2TZ, UK

**Keywords:** Fasting, Functional feeds, Plant proteins, Vegetable oils, Gut inflammation, Transcriptomics

## Abstract

The interplay between nutrition and immune system is well recognised, however the true integration of research between nutrition, animal energy status and immune function is still far from clear. In fish nutrition, especially for species maintained in aquaculture, formulated feeds are significantly different from the natural diet with recent changes in nutrient sources, especially with protein and oil sources now being predominated by terrestrial derived ingredients. Additionally, many feeds are now incorporated to health management and termed functional feeds, which are believed to improve fish health, reduce disease outbreaks and/or improve post-infection recovery. Using new omics technologies, including transcriptomics (microarray and RNA-seq) and proteomics, the impacts of nutrition on the immune system is becoming clearer. By using molecular pathway enrichment analysis, modules of genes can indicate how both local (intestinal) and systemic immune function are being altered. Although great progress has been made to define the changes in host immune function, understanding the interplay between fish nutrition, intestinal microbiome and immune system is only just beginning to emerge.

## Highlights

• Nutrition has direct impacts on fish health and immunity.
• Omics technologies are essential for understanding diet and immunity interactions.
• Omics approaches help to improve functionality and sustainability of aquaculture feeds.
• Future research should focus on microbiota, early life experience and adaptive immunity.

## 1 Introduction

Both energy and nutrients consumed with food are essential for maintaining optimal immune function (Chandra, 1997). Without adequate nutrition, the immune system is deprived of the resources that are needed to defend the host against bacteria, viruses and parasites. According to the epidemiological and clinical studies, nutritional deficiencies substantially alter immunocompetence and increase the risk of infection (Marcos et al., 2003). Human malnutrition is usually a complex syndrome of multiple nutrient deficiencies, caused by insufficient intakes of energy, macronutrients and/or micronutrients. However, even a single nutrient deficiency (such as lack of specific vitamins, minerals or trace elements) may have detrimental effects on functioning of immune system, as demonstrated in laboratory rodents during the feeding trials (Beck and Matthews, 2000, Blewett and Taylor, 2012). Understanding the impacts of adequate nutrition on immunity is important not only for human health, but also for the increasing number of animals maintained on formulated diets.

Recent interest in fish nutrition has been fuelled by the rapid expansion of aquaculture industry, with worldwide fish production continuing to increase at approximately 5% per year (Food and Agriculture Organization of the United Nations, FAO, 2016). Despite nearly 370 fish species (including hybrids) being registered by FAO as cultured commercially, diet formulations for many of these species are based on limited information about their nutrient requirements (Lall, 2000, Hamre et al., 2013). Farmed fish are also commonly subjected to short-term food deprivation (fasting) as part of a seasonal feeding pattern and in response to overproduction or disease outbreaks, yet fasting-induced impacts on fish immunity are largely unknown and differ between species (Li et al., 2014). Further complexity is added by the global decrease in the availability of high quality marine ingredients for aquaculture feeds, such as fish meal as a protein source and fish oil as a lipid source (Naylor et al., 2000, Bostock et al., 2010, Tocher and Glencross, 2015, Jobling, 2016). These two ingredients are of great importance as they supply essential amino acids (such as lysine and methionine) that are often deficient in plant proteins and fatty acids (eicosapentaenoic acid (EPA) and docosahexanoic acid (DHA)) that are not found in vegetable oils. With the wild fish supplies being unable to meet the demand from aquaculture, farmed fish are increasingly exposed to dietary plant materials, often without a comprehensive understanding of their impacts on fish health and ability to resist pathogens.

Fish have considerably higher exposure to pathogens than non-aquatic vertebrates, with typically a million of bacteria and 10 million of viruses per millilitre of seawater, including both pathogenic and non-pathogenic forms (Fuhrman, 1999). The pathogen exposure in fish starts immediately after hatching from their protective chorions and is further enhanced during the mouth and gut opening stages and at the onset of exogenous feeding (Castro et al., 2015). Furthermore, many fish species are exposed to different and unfamiliar pathogens when they switch between fresh and salt water environments (Jeffries et al., 2014). Evidence is also growing that some fish, including non-migratory species, are being exposed to novel pathogens as a result of climate change (Crozier and Hutchings, 2014). Finally, outbreaks of fish diseases commonly occur when fish are stressed due to a variety of factors associated with the aquaculture environment and management procedures such as high stocking densities, transport or handling. Such stress may induce a variety of physiological responses grouped broadly as primary (release of adrenal catecholamines and corticosteroids) and secondary, which include changes in energy metabolism and hydromineral balance, coupled with alterations in cardiovascular, respiratory and immune functions (Barton, 2002). Both primary and secondary stress responses may then contribute to the changes in whole-animal performance (called tertiary stress responses), most likely by redirecting energy and other resources from one set of physiological processes to another, resulting for example in impaired resistance to disease and enhanced pathology during infection (Vargas-Chacoff et al., 2016). Among the most common causative agents of infectious diseases in aquaculture are bacteria (54.9%), followed by viruses (22.6%), parasites (19.4%) and fungi (3.1%) (McLoughlin and Graham, 2007). Because the ability of fish to resist pathogens and cope with stress depends to a large extent on their nutritional status, the need for dietary interventions that would improve fish health has become widely recognised as central to sustainable aquaculture and the future of industry.

In fish as in other vertebrates, immunity is typically divided into two distinct components: the innate immune response and the adaptive immune response. Innate immunity is the first line of defence against infection and it includes both physical barriers as well as humoral and cellular responses. The adaptive immune response also relies on humoral and cellular mechanisms and is characterised by a specific antigen recognition that drives a stronger and faster secondary pathogen-specific immune response. Recent advances in general and fish immunology have demonstrated that many of the cells and molecules considered unique to either the innate or adaptive systems play specific roles in both of them, making the cross-talk between the innate and adaptive systems more complex than previously thought (Secombes, 2016). The cellular components of fish immunity include T and B lymphocytes, natural killer cells, monocytes, macrophages, neutrophils, eosinophils, mast cells and thrombocytes (Castro and Tafalla, 2015) as well as dendritic-like cells described recently in Atlantic salmon (*Salmo salar*) (Fuglem et al., 2010, Haugland et al., 2012), rainbow trout (*Oncorhynchus mykiss*) (Johansson et al., 2012, Granja et al., 2015) and zebrafish (*Danio rerio*) (Lugo-Villarino et al., 2010). While in some tissues (such as gonads) the population of leukocytes is relatively sparse, substantially more immune cells are found in mucosal tissues such as gut, gills, and skin. Consequently, these structures are classified as gut-associated lymphoid tissue (GALT), gill-associated lymphoid tissue (GIALT) and skin-associated lymphoid tissue (SALT).

The mucosal layers of GALT, GIALT and SALT interfere with pathogens not only by trapping them, but also through the action of a variety of antimicrobial factors present in the mucus like lectins, lysozymes, pentraxins, complement proteins, antibacterial peptides and immunoglobulins (Salinas and Miller, 2015). If, however, the pathogen succeeds to penetrate the mucosal epithelium, it encounters the innate cellular machinery, triggered by the cell types equipped with invariable receptors called pattern recognition receptors (PRRs), able to recognize common conserved molecules (PAMPs) characteristic of many microbial agents (Castro and Tafalla, 2015). The uptake of the antigen then leads to 1) release of cytokine mediators and attractants for different cell types to initiate the inflammatory process, and 2) antigenic presentation through the major histocompatibility complex (MHC) in the lymphoid tissues for the activation of the primary responses of antigen-specific lymphocytes expressing variable receptors able to recognize molecules specific to the pathogen, thus contributing to the development of secondary responses and memory. Due to the extensive interplay between innate and adaptive immunities, both these systems are according to the human studies equally sensitive and responsive to nutritional deficiencies, with the most consistent abnormalities seen in cell-mediated immunity, complement system, phagocyte function, cytokine production, mucosal secretory antibody response and antibody affinity (Marcos et al., 2003).

**Fig. 1:**
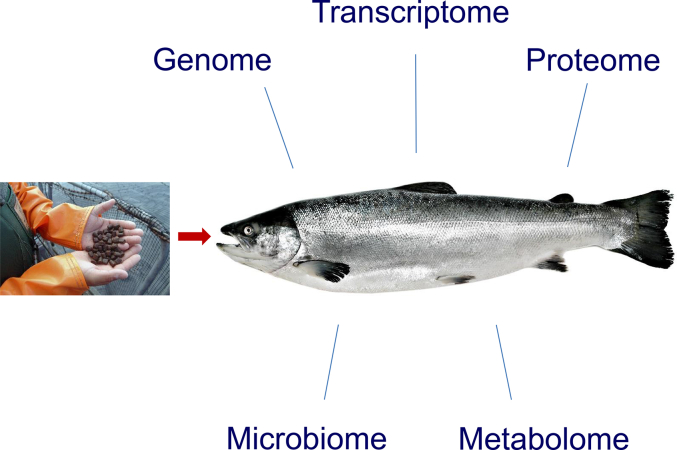
Once feed is digested and absorbed, nutrients and feed additives influence gene activation and transcription, protein expression, enzyme activities, metabolism as well as gut microbial community (microbiota) and its component genes (microbiome). Gene expression profiling (transcriptomics) along with monitoring of protein expression (proteomics) and metabolites (metabolomics), coupled with microbiome profiling (microbiomics), provide holistic overviews of these diet-induced changes and their impacts on fish health and immunity.

In this review, we will present the current use of high-throughput omics approaches to investigate the interplay between nutrition and immunity in fish (Fig. 1). Specifically, we will focus on recent advances in omics technologies, which include high-throughput transcriptomics by both microarray and RNA-seq approaches, but also proteomics examining the final products of gene expression. The advantages and disadvantages of the transcriptomic approaches have been discussed previously (Martin et al., 2016). The use of high-throughput techniques allows gene networks and molecular pathways to be identified that would not be possible when investigating only a small number of genes at any one time. There are advances being made using other technologies for deeper understanding of gene regulation including non-coding RNAs, epigenetics and metabolomics, however at the time of writing this review, these approaches have not been used to examine the relationship between nutrition and immune function and will not be covered here.

## 2 Overview of genomic resources for aquaculture

One of the central criteria for successful high-throughput genomics is the availability of genomic resources. With the dramatic increase in availability of whole genomes and the reduced cost of deep sequencing, the landscape is changing at an increasing speed (Abdelrahman et al., 2017). Until several years ago, there were only genome sequences for a limited number of model species such as zebrafish (Howe et al., 2013) and pufferfish (*Fugu rubripes*) (Aparicio et al., 2002), from which inferences to non-model aquaculture species could be made. However at the time of writing this review, there are 82 fish genomes available on NCBI, 14 of these genomes are now anchored to chromosomes, and the remaining being at various stages draft sequences yet to be fully assembled. The implication is that there is a vast resource for gene and protein expression studies available for farmed fish. The key aquaculture species including Atlantic salmon (Lien et al., 2016), rainbow trout (Berthelot et al., 2014), Atlantic cod (*Gadus morhua*) (Star et al., 2011), common carp (*Cyprinus carpio*) (Xu et al., 2014), European sea bass (*Dicentrarchus labrax*) (Tine et al., 2014), tilapia (*Oreochromis niloticus*) (Brawand et al., 2014), grass carp (*Ctenopharyngodon idellus*) (Wang et al., 2015) and channel catfish (*Ictalurus punctatus*) (Liu et al., 2016) are examples of completed genomes, which have also gene models and RNA-seq resources to define the transcriptome. These resources are being continually annotated to a greater depth and allow for direct mapping of RNA-seq and gel-free proteomic mass spectrometry outputs to be achieved.

## 3 Overview of dietary manipulations affecting fish immunity

### 3.1 Fasting

In nature, fish can undergo extended periods of fasting, often associated with spawning migrations, during which some species of salmonids can go without food for many months (Mommsen, 2004). Yet, there are only few studies that have specifically examined the relationship between fasting and immune function using omics approaches (Table 1). The Atlantic salmon liver transcriptome was examined following infection with a bacterial pathogen *Aeromonas salmonidica*, with groups of fish being either fed or starved prior to infection (Martin et al., 2010). Microarray analysis clearly demonstrated that in the uninfected starved fish compared to the uninfected fed fish, there was a clear decrease in many components of the immune system, suggesting down-regulation of constitutive immune genes, most likely as an energy conserving mechanism. However, when the fish were infected, the magnitude of response of many acute phase response proteins was greater in the starved fish than in fed fish. The likely reason for this is that the starved fish were compensating and attempting to produce sufficient mRNAs for immune protein transcription. A more recent study involved the impact of 7-day starvation on channel catfish fingerlings. These fish are known to become highly vulnerable to *Flavobacterium columnare*, when undergoing food deprivation. The skin and gills were subjected to RNA-seq analysis to gain insights into mucosal immune system following starvation. Overall, the RNA-seq showed the majority of transcripts to be down-regulated and within these, a core group could be associated with immune function, including components of the Toll pathway, iNOS, lysozyme and peptidoglycan recognition protein 6. A number of complement genes were decreased in expression as were chemokines and interleukins 17 and 22. Together, these two studies show how transcriptomics reveals the immune components that are affected even by a short-term food withdrawal and as such feeding regimes need to be carefully assessed not only during commercial production but also when laboratory-based challenge experiments are being performed.

**Table 1:** Fish studies using omics technologies to evaluate effects of fasting on immunity.

Fish species	Omics technology	Dietary manipulation	Disease challenge	Comparison and sampling	Tissue analysed	Main findings	Reference
Atlantic salmon (*Salmo salar*)	Transcriptomics (microarray)	Fasting	*Aeromonas salmonicida*	Fish fasted for 4 weeks and control non-fasted fish were injected with pathogen or PBS, sampled 24 h post-infection	Liver	Fasting reduced expression of immune genes and altered response of liver transcriptome to infection	Martin et al., 2010
Atlantic salmon (*Salmo salar*)	Transcriptomics (microarray)	Caloric restriction of fish meal (FM-CR, 40% of ad libitum food intake) to mimic reduced intake of soybean meal (SBM)	–	Fish fed FM-CR vs SBM vs control FM (54 d)	Liver and distal intestine	CR and SBM altered expression of both pro- and anti-inflammatory genes	Skugor et al., 2011
Channel catfish (*Ictalurus punctatus*)	Transcriptomics (RNA-seq)	Fasting	–	Fish fasted for 7 d vs control non-fasted fish	Gills and skin	Fasting significantly altered expression of critical innate immune factors in a manner consistent with lower immune fitness as well as dysregulating key genes involved in energy metabolism and cell cycling/proliferation.	Liu et al., 2013
Blue catfish (*Ictalurus furcatus*)	Transcriptomics (RNA-seq)	Fasting	–	Fish fasted for 7 d vs control non-fasted fish	Gills and skin	Fasting perturbed arginine synthesis and metabolism pathways in a manner likely altering macrophage activation states and immune readiness	Li et al., 2014

### 3.2 Functional feeds

Functional feeds are defined as feeds which are supplemented to enhance the health benefits to the fish, with the additives being beyond the basal requirements of the fish for normal growth and health. Such diets encompass many different components including micro ingredients such as selenium, zinc and vitamins, other components that act as immunostimulants such as algal and plant extracts are now often utilized. Prebiotics which are often derived from yeast extracts are believed to enhance the functionality of the microbiome in the fish intestine (Hoseinifar et al., 2016), whereas probiotics are bacterial species added directly to the diet with the aim of these becoming established in the intestine (Merrifield et al., 2010, Carbone and Faggio, 2016, Dawood and Koshio, 2016). Additional functional ingredients include oils such as high marine oil diets or phospholipid rich diets derived from krill that may enhance the immune function (Martinez-Rubio et al., 2013). Such diets are under continued development and increasingly viewed as important complementary component of fish health management in aquaculture (Tacchi et al., 2011). The expectation is that these diets will enhance the innate immune system enabling the fish to better resist pathogens such as sea lice in Atlantic salmon, or enable faster clearance of pathogen such as viruses once infected. There is growing literature on such feed additives (e.g., Song et al., 2014), however the mechanisms by which these feeds alter metabolism and immune capacity is poorly understood. Nevertheless, evidence is growing that these dietary interventions do have an impact on both local (intestinal) and systemic immune systems (Lazado and Caipang, 2014). A further use for functional feeds in aquaculture has been to alleviate the impacts of high levels of antinutritional factors (ANFs) that are not fully removed from plant-derived proteins. Omics approaches have the potential to define some of the responses to such diets by allowing a holistic view of the transcriptome and proteome to be examined at once (Table 2).

**Table 2:** Fish studies using omics technologies to evaluate effects of functional feeds on immunity.

Fish species	Omics technology	Dietary manipulation	Disease challenge	Comparison and sampling	Tissue analysed	Main findings	Reference
Atlantic salmon (*Salmo salar*)	Transcriptomics (RNA-seq)	Supplementation with in-feed plant-derived additives (1% immunostimulant and 3% of anti-attachment compound)	Sea lice (*Caligus rogercresseyi*)	Fish fed supplemented and control diets for 21 days were infected with parasite, sampled 15 days post-infection	Skin and head kidney	In-feed additives decreased lice infection and altered expression of immune genes (including MHC-I and MHC-II transcripts), suggesting improved immunity	Núñez-Acuña et al., 2015
Atlantic salmon (*Salmo salar*)	Transcriptomics (microarray)	Supplementation with glucosinolates (GLs, 7.3 μmol/g and 26.4 μmol/g)	Salmon lice (*Lepeophtheirus salmonis*)	Fish fed high dose of GLs for 17–18 d vs control diet, sampled prior to infection; Fish fed high dose of GLs, low dose of GLs and control diet were infected with parasite, sampled 5 weeks post-infection	Liver, distal kidney and muscle	GLs decreased lice infection and increased expression of genes associated with iron and heme withdrawal response, supporting hypothesis that making heme unavailable to lice could be part of an effective anti-parasitic strategy	Skugor et al., 2016
Atlantic salmon (*Salmo salar*)	Transcriptomics (microarray)	Supplementation with glucosinolates (GLs, 7.3 μmol/g and 26.4 μmol/g)	Salmon lice (*Lepeophtheirus salmonis*)	Fish fed high dose of GLs for 17–18 d vs control diet, sampled prior to infection; Fish fed high dose of GLs, low dose of GLs and control diet were infected with parasite, sampled 5 weeks post-infection	Skin	GLs decreased lice infection, increased expression of IFN-related genes prior to infection and induced higher expression profiles of Type 1 immune genes late into the infection	Jodaa Holm et al., 2016
Atlantic salmon (*Salmo salar*)	Transcriptomics (microarray)	Supplementation with a number of additives, including nucleotides, mannooligosaccharides, fructooliogsaccharides, vitamin C and vitamin E	–	Fish fed supplemented vs control diet (16 weeks)	Liver and muscle	Supplemented diet reduced hepatic expression of genes encoding proteins involved in innate and adaptive immune responses	Tacchi et al., 2011
Atlantic salmon (*Salmo salar*)	Transcriptomics (microarray)	Two functional feeds (FF1 and FF2) with reduced levels of total lipid and digestible energy, and different levels and proportions of long-chain polyunsaturated fatty acids (LC-PUFA)	Atlantic salmon reovirus (ASRV) associated with heart and skeletal muscle inflammation (HSMI)	Fish fed FF1, FF2 and control diet for 10 weeks were infected with pathogen, sampled 8, 10, 12 and 16 weeks post-infection	Heart	FF1 and FF2 reduced viral load and severity of heart lesions, and greatly affected expression of inflammation/immune related genes over the course of ASRV infection	Martinez-Rubio et al., 2012
Atlantic salmon (*Salmo salar*)	Transcriptomics (microarray)	Two functional feeds (CMS1 and CMS2) with reduced lipid content and increased eicosapentaenoic acid (EPA) levels	Piscine Myocarditis Virus (PMCV) associated with cardio myopathy syndrome (CMS)	Fish fed CMS1, CMS2 and control diet for 10 weeks were infected with pathogen, sampled 6, 8 and 14 weeks post-infection	Heart	CMS1 and CMS2 reduced viral load and severity of heart lesions, and greatly affected expression of inflammation/immune related genes, leading to a milder and delayed inflammatory response over the course of PMCV infection	Martinez-Rubio et al., 2014
Atlantic salmon (*Salmo salar*)	Transcriptomics (microarray)	Four diets (D1-D4) with increasing levels of n-3 LC-PUFA-rich microalgae (0, 1, 6 and 15 g/kg)	–	Fish fed D2-D4 vs control D1 diet (12 weeks)	Liver	Supplemented diet altered expression of genes involved in innate immune responses	Kousoulaki et al., 2015
Atlantic salmon (*Salmo salar*)	Transcriptomics (microarray)	Five diets (D1-D5) with increasing levels of docosahexaenoic acid (DHA, 1, 3, 6, 10 and 13 g/kg)	–	Fish fed D2-D5 vs control D1 diet (62 d)	Liver	Increasing levels of dietary DHA were associated with upregulation of immune pathways, especially chemokine signalling, FC epsilon RI signalling and natural killer cell mediated cytotoxicity pathways	Glencross et al., 2015
Rainbow trout (*Oncorhynchus mykiss*)	Transcriptomics (microarray)	Two diets with low levels of phosphorus (LP, 0.15%) and sufficient levels of phosphorus (SP, 0.60%)	–	Fish fed LP vs SP (20 days)	Proximal intestine	LP diet inhibited the expression of interferon-inducible genes involved in immune responses against viruses, suggesting reduced immunity	Kirchner et al., 2007
Rainbow trout (*Oncorhynchus mykiss*)	Transcriptomics (RNA-seq)	Supplementation with a vitamin and mineral premix	–	Fish fed supplemented vs micronutrient deficient diet (10 weeks)	Liver	Micronutrient deficient diet impacted transcriptional factors related to cellular metabolism, functions and structures, and altered genes associated with negative acute phase response proteins	Olsvik et al., 2013
Rainbow trout (*Oncorhynchus mykiss*)	Transcriptomics (microarray)	Three diets (D1-D3) with increasing levels of Sel-Plex^®^ (selenium supplementation at 0, 1 and 4 mg/kg)	Polyinosinic:polycytidylic acid (poly(I:C)), a pathogen-associated molecular pattern (PAMP) that mimics viral infection	Fish fed supplemented (D2-D3) and control diet (D1) for 10 weeks were injected with poly(I:C) or PBS, sampled 24 h post-injection	Head kidney and liver	D3 diet increased expression of several genes associated with antiviral defences (especially IFN-γ and downstream molecules involved in cell-mediated immune response), suggesting improved immunity	Pacitti et al., 2016
Channel catfish (*Ictalurus punctatus*)	Transcriptomics (RNA-seq)	Supplementation with Actigen^®^ (a yeast mannan oligosaccharide (MOS) product)	*Flavobacterium columnare*	Fish fed supplemented and control diet for 9 weeks were infected with pathogen, sampled prior to infection (0 h) and 8 h post-infection	Gills	MOS altered mannose receptor DEC205 and IL4 signalling at 0 h, and then reduced expression of inflammatory cytokines, shifting response patterns to favour resolution and repair (8 h post-infection)	Zhao et al., 2015

Sea lice are a major parasitic pest of Atlantic salmon and are represented by two main species 1) *Lepeophtheirus salmonis*, which is currently the most abundant species in the northern hemisphere salmon aquaculture, and 2) *Caligus rogercresseyi*, which is the predominant species impacting salmon farming in Chile. Varying levels of resistance to diets containing emamectin benzoate (Covello et al., 2012, Aaen et al., 2015) have brought a new emphasis on lice control, as costs for treatments are continually growing (Burridge et al., 2010). Lice cause immunomodulation in the skin and mucosal surface by immunosuppression, resulting in a TH2 type of response (Skugor et al., 2008, Tadiso et al., 2011). Resistant salmon show a greater Th1 and Th17 response, which is believed to drive early protection to lice infection (Fast, 2014, Braden et al., 2015). Both management and control of sea lice numbers have direct and indirect impacts on the salmon health, and functional feeds are viewed as a potential part of integrated pest management for these parasites in parallel to fallowing, choice of farm site, chemotherapeutic and selection for resistance lines of salmon (Tsai et al., 2016). Dietary additives have impacts on lice settlement (Jensen et al., 2015), however to improve and understand the mechanisms by which these diets cause reduced infestation, the use of omics approaches would be beneficial to employ, in particular to determine if there are associated alteration in the immune system, or if the effects are exerted by other routes.

Among the dietary additives important for sea lice control are glucosinolates, When these plant derived-phytochemicals are metabolised by fish intestinal microbiota to isthiocynates, they can generate anti-inflammatory responses and promote antioxidant status and detoxification, but they may also promote pro-inflammatory responses in species such as mice. Diets containing glucosinolates were shown to reduce sea lice burden by up to 25% in comparison to control diets (Jodaa Holm et al., 2016). The transcriptome analysis of skin showed that interferon and related genes were increased in expression by the supplemented diet prior to infection, and that supplementation induced higher expression profiles of Type 1 immune genes late into the infection. It was of interest that many of the transcripts associated with the lice burden were antiviral, but also chemokines and genes associated with acute phase response were involved in preventing the immunosuppressive effect of the lice. The fish however may also perceive glucosiolates as toxins and further transcriptomics investigations to liver, kidney and muscle showed significant enrichment for detoxification genes in the liver, but consistent with skin discussed above the kidney showed increased in interferon genes (Skugor et al., 2016), with other processes including iron metabolism being altered, which has been associated with lice resistance (Sutherland et al., 2014). Another plant-derived functional feed with anti-lice properties has been described by Núñez-Acuña et al. (2015), with lice counts significantly reduced following the feeding trial. The RNA-seq was carried out on both skin and head kidney, however no details of the dietary component have been given, so it is impossible to interpret the results in the context of potential mechanisms.

Beta-glucans (β-G) and mannan oligosaccharides (MOS) are prebiotics commonly used in fish and are naturally occurring indigestible carbohydrates found in the yeast cell wall (YCW). Both β-G and MOS have been implicated in reduced sea lice load (Refstie et al., 2010) as well as improved response to other pathogens. Several proteomics studies that have examined skin and mucus proteomes suggest that the supplemented diets are modulating mucus to prevent lice attachment, with a variety of different proteins being associated with immune function such as lysozyme and galactins, which bind carbohydrates (Provan et al., 2013). A further study using YCW indicated calreticulin, (a multifunctional protein that occurs in the endoplasmic reticulin and plays a critical role in protein folding and degradation of glycoproteins) as a putative key mucosal protein induced by the YCW feed (Micallef et al., 2017), however a direct link to how this protein affects development of mucosal immune response is not yet established. Diets containing YCW are also associated with increased survival of channel catfish when challenged with *Flavobacterium columnare*, the causative agent of columaris disease. During pre-feeding phase, the impact of these diets on gill, the major route of infection, was examined by RNA-seq showing the changes in the mannose receptor and genes related to IL4 signalling, which may modify the host response to the infection (Zhao et al., 2015). These results suggested that IL4 signalling could reduce the pro-inflammatory response and drive tissue repair and enable resolution of the infection, with a Th2/M2 macrophage response being associated with the increased survival in fingerling catfish. This finding corroborates the hypothesis that lectins (especially the rhamnose binding lectins) and cytokines associated with IL4 R are implicated in the resistance to bacterial attachment (Peatman et al., 2013). Lactocellulose, another indigestible carbohydrate synthetized from sucrose and lactose, has been used as a prebiotic in grass carp (*Ctenopharyngodon idella*), after which the liver transcriptome was assessed using a zebrafish microarray (Chu et al., 2013). It was suggested that immune function in liver was modulated by increased expression of novel immune receptors, leucocyte derived chemotaxins and α 2 macroglobulin, yet the specificity of hybridization to the array is uncertain even though both species are cyprinids.

With the diets of farmed piscivourous fish changing dramatically in recent years, attempts have been made to manipulate the lipid sources to modulate the outcome of infection and post-infection tissue repair. Eicosanoids derived from arachidonic acid can promote inflammation, whereas eicosanoids formed from docosahexaenoic acid and eicosapentaenoic acid generally reduce inflammation and hence reduce the overall damage caused by the inflammatory process. Although fish fed high levels of vegetable oil grow well, these oils may have negative impacts on the ability to mount the correct response to infection under stressful condition and exposure to multiple pathogens. Two viral diseases in Atlantic salmon, heart and skeletal muscle inflammation (HSMI) and cardiac myopathy syndrome (CMS) are chronic diseases that result in viral accumulation in the heart and subsequent cardiac damage. Diets rich in fish oil and also krill oil (which is especially rich in phospholipids) were compared to a standard salmon diet containing high levels of rapeseed oil. Following infection with piscine reovirus (PRV), fish fed the diets rich in marine oils showed lower viral replication and subsequently less pathology in the heart, probably due to a dampened inflammatory response (Martinez-Rubio et al., 2012). Microarray analysis of heart tissue from selected time points following infection clearly demonstrated the reduced expression of key groups of immune genes until 12 weeks post-infection, after which there was a marginal rise in antiviral genes and antigen presentation, suggesting improved clearing of the virus. Another trial examining marine lipid functional feeds (specifically enriched with EPA) and responses to CMS showed similar mechanisms occurring (Martinez-Rubio et al., 2014), with antiviral and interferon genes generally suppressed as well as reduced stimulation of inflammatory genes following infection as compared to fish fed the reference diet. These experiments show that the lipid source can have major implications for disease response, which is typically not observed during normal growth. Further evidence for DHA having direct impacts on immune transcripts was observed following a graded feeding trial in salmon (Glencross et al., 2015). This study demonstrated an increased expression of genes associated with immune system in the liver with gene sets enriched for chemokine signalling and natural killer cell mediated toxicity, but no disease challenge was performed and the data were not fully interrogated to examine immune function. Functional feeds containing phospholipids have also been shown to improve the survival outcomes for sea bream during cold water conditions that cause “winter disease”, a non-specific decrease in metabolic function and suppressed immunocompetence and performance (Tort et al., 1998). Furthermore, diets rich in fish oil and krill oil have also been shown to improve the survival rates of fish suffering from winter disease, while recent proteomic studies in both plasma (Schrama et al., 2016) and liver (Richard et al., 2016) identified proteins indicative of an improved immune status, improved cellular stress response and altered lipid metabolism.

Feed supplements can include vitamins and minerals, however to date there has been little research using vitamins in fish and employing omics approaches. As for micronutrients, recent work on selenium (Se) has demonstrated that this element can impact on immune responses. Selenium is an essential micronutrient and different chemical forms can have differing bioavailability. Organic selenium such as yeast derived Sel-plex^®^ (manufactured by Alltec) has higher bioavailability than inorganic Se (Pacitti et al., 2015) and may be used to increase Se levels in diets without inducing any toxic effects. Sel-plex enriched diets were feed to rainbow trout, after which the antiviral responses in head kidney and liver were assessed following a poly I:C stimulation (mimicking a viral infection). Transcriptomic profiling was performed by microarray and this clearly showed an enhance response to the stimulant in the fish fed Se rather than the basal diet (Pacitti et al., 2016). The response was much greater in the head kidney than in liver, in particular interferon γ and downstream proteins involved in cell mediated immunity appeared to be enhanced in expression by the Se-enriched diets. These results show that the capacity and components of the immune system can only be fully assessed by high-throughput transcriptomics rather than the single gene approach.

### 3.3 Use of plant materials to replace marine ingredients

Most fish need relatively high levels of protein and oil in their diet as they do not utilise carbohydrates efficiently enough to rely on them as a source of energy (De Silva and Anderson, 1995). For this reason, carnivorous, herbivorous and omnivorous fish all require similar quantities of dietary protein and oil per body weight, but it is the group of carnivorous fish such as salmonids that need the highest levels of fish meal and fish oil to ingest, as these marine ingredients closely resemble the natural feeding habits of predatory fish (Lovell, 1998). As a result, farmed salmonids are highly sensitive to the dietary changes that involve replacement of marine ingredients with alternative plant materials, but these dietary shifts are inevitable and are expected to continue into the future (Naylor et al., 2009, Hardy, 2010). Indeed, the global contribution of fish meal to salmon feeds has decreased from ∼45% in 1995 to ∼20% in 2012 and is predicted to reduce further to ∼12% by 2020 (Bostock et al., 2010). Similarly, the global use of fish oil in salmon feeds has decreased from ∼25% in 1995 to ∼12% in 2012 and is expected to drop to ∼8% by 2020 (Bostock et al., 2010). Understanding the impacts of plant materials on fish health has been greatly advanced by employing the omics technologies to determine the molecular and cellular aspects of immune responses in organs directly affected by diet, such as intestine and liver.

#### 3.3.1 Plant proteins

Sources of dietary protein have changed dramatically in the last decade with reduced reliance on marine derived proteins and greater use of terrestrial protein sources (Ytrestøyl et al., 2015, Jobling, 2016). This change although ensuring good growth is not without its complications and complex interaction with host microbiome and immune system. There are two key factors regarding plant proteins in fish feed. Firstly, there is imbalance of essential amino acids (Jobling, 2016) with lysine and methionine often being below the required levels for fish. Secondly, plant defence molecules (ANFs) can be co-purified with the proteins. Essential amino acids in the form of crystalline amino acids can be added to the diet at a cost, and also “functional amino acids” can be used to enhance specific aspects of physiology and health such as arginine (Jiang et al., 2015). However, the problems generated by the presence of ANFs in aquaculture feeds are typically more difficult to address. When ingested, these ANFs impact on digestion, absorption and utilisation of nutrients and negatively modify intestinal physiology (Krogdahl and Bakke, 2015). The major plant materials used in aquaculture feeds are legumes such as beans, lupins and peas, which are rich in digestible proteins and have favourable amino acid profiles (Hardy, 2010). The ANF content varies between species and depends on the method used for protein extraction (Champ, 2002). Soybean meal (SBM), a relativity crude protein extract can induce gut inflammation (enteritis) in the distal intestine (Baeverfjord and Krogdahl, 1996). Intestinal transcriptome profiling has been carried out independently by a number of groups that indicate conserved responses to these diets (Table 3). Early alterations of the gut transcriptome were examined by Sahlmann et al. (2013), indicating a rapid response to the dietary components. However, many of these changes were still present after 8–10 weeks of feeding with SBM (De Santis et al., 2015, Król et al., 2016), indicating that clear immune responses were followed by dysfunction of intestinal barrier and unresolved gut inflammation.

**Table 3:** Fish studies using omics technologies to evaluate effects of plant proteins on immunity.

Fish species	Omics technology	Dietary manipulation	Disease challenge	Comparison and sampling	Tissue analysed	Main findings	Reference
*Plant proteins used to replace fish meal*							
Atlantic salmon (*Salmo salar*)	Transcriptomics (microarray)	2 diets with soy protein concentrate (SPC) and fish meal (FM)	–	Fish fed SPC vs FM diet (77 d)	Mid intestine, liver and skeletal muscle	SPC diet altered expression of immune genes in mid intestine (most genes upregulated, some downregulated), liver (most genes downregulated) and skeletal muscle (most genes downregulated), indicating both local and systemic immune responses to SPC, despite unchanged organ histology	Tacchi et al., 2012
Atlantic salmon (*Salmo salar*)	Transcriptomics (microarray)	5 plant protein (PP) diets supplemented with soyasaponin; PP included corn gluten, pea protein concentrate, sunflower, rapeseed and horsebean (non-supplemented PP diets were used as controls)	–	Fish fed supplemented vs non-supplemented diets (80 d)	Distal intestine	Combination of pea protein concentrate and soyasaponin induced gut inflammation and altered expression of immune genes (up-regulation of cytokines, NFkB and TNFalpha related genes and regulators of T-cell function, coupled with down-regulation of IFN-axis)	Kortner et al., 2012
Atlantic salmon (*Salmo salar*)	Transcriptomics (microarray)	2 diets with 20% soybean meal (SBM) and fish meal (FM)	–	Fish fed SBM vs FM diet (1, 2, 3, 5 and 7 d)	Distal intestine	SBM diet induced gut inflammation at histological level and increased expression of immune-related genes, including GTPase IMAP family members, NF-kB-related genes and regulators of T cell and B cell function, indicating a rapid onset of disease	Sahlmann et al., 2013
Atlantic salmon (*Salmo salar*)	Transcriptomics (microarray)	4 diets with 0, 10, 20 and 30% soybean meal (SBM)	–	Fish fed 10, 20 and 30% SBM vs 0% SBM (12 weeks)	Distal intestine and liver	Diet with 30% SBM altered expression of immune genes in distal intestine (pathways associated with phagocytosis and antigen processing and presentation) and liver (up-regulation of several genes of the complement cascade), suggesting both local and systemic inflammatory responses to SBM	De Santis et al., 2015
Atlantic salmon (*Salmo salar*)	Transcriptomics (microarray)	6 plant protein (PP) diets and control fish meal (FM) diet; PP included bean (BPC) and soy (SPC) protein concentrates and soybean meal (SBM)	–	Fish fed PP vs FM diet (56 d)	Distal intestine	High levels of PP inclusion in 36% SBM and 45% BPC diets induced gut inflammation at histological level and altered pathways associated with inflammatory and immune responses, suggesting ongoing disease	Król et al. 2016
Zebrafish (*Danio rerio*)	Transcriptomics (RNA-seq)	2 diets with high (HNPM) and low (LNPM) novel protein meal	–	Fish fed HNPM vs LNPM diets from 11 to 21 d post-fertilization	Intestine	HNPM diet affected limited number of immune-related genes, including up-regulation of stanniocalcin 1, interlectin 2, radical S-adenosyl methionine domain containing 2, ISG15 ubiquitin-like modifier and B cell CLL/lymphoma 6a	Rurangwa et al., 2015
*Supplements used to prevent SBM-induced gut inflammation*							
Atlantic salmon (*Salmo salar*)	–	Supplementation of 20% soybean meal (SBM) with bacteria meal (BM, 300 g/kg); fish meal (FM), non-supplemented 20% SBM and BM diets were used as controls	–	Fish fed SBM-BM vs FM, SBM and BM diets (80 d)	Distal intestine	SBM-BM diet prevented gut inflammation, probably by normalising intestinal barrier function	Romarheim et al., 2011
Atlantic salmon (*Salmo salar*)	Transcriptomics (microarray)	Supplementation of 20% soybean meal (SBM) with either one of three yeasts *Candida utilis* (CU), *Kluyveromyces marxianus* (KM), *Saccharomyces cerevisiae* (SC) or microalgae *Chlorella vulgaris* (CV); non-supplemented 20% SBM and fish meal (FM) were used as controls	–	Fish fed supplemented vs FM and SBM diets (4 weeks)	Distal intestine	CV and CU diets prevented gut inflammation by normalising expression of genes 1) associated with NOD-like receptor signalling and chemokine signalling pathways and 2) encoding antimicrobial peptides	Grammes et al., 2013
Atlantic salmon (*Salmo salar*)	–	Supplementation of 20% soybean meal (SBM) with bacteria meal (BM, 25, 50, 100, 150, 200 and 300 g/kg); fish meal (FM) and non-supplemented 20% SBM diets were used as controls	–	Fish fed supplemented vs FM and SBM diets (47 d)	Distal intestine	SBM-BM diets prevented gut inflammation in a dose dependent manner, by normalising numbers of 1) cluster of differentiation 8 α positive (CD8α^+^) intraepithelial lymphocytes and 2) MHC II-reactive cells	Romarheim et al., 2013a
Atlantic salmon (*Salmo salar*)	–	Supplementation of 20% soybean meal (SBM) with basic bacteria meal (BM), autolyzed BM (AUT), permeate (PER) or retentate (RET) from filtration of AUT, nucleic acid reduced *M. capsulatus* (MCap), and *M. capsulatus* grown on methanol (MeOH); fish meal (FM) and non-supplemented 20% SBM diets were used as controls	–	Fish fed supplemented vs FM and SBM diets (4 weeks)	Distal intestine	SBM-BM diets prevented gut inflammation in a dose dependent manner, by normalising numbers of 1) cluster of differentiation 8 α positive (CD8α^+^) intraepithelial lymphocytes and 2) MHC II-reactive cells	Romarheim et al., 2013b
Atlantic salmon (*Salmo salar*)	–	Supplementation of low fish meal (LFM) diet and high protein soybean meal (HPS) diet with various bile components and lecithin	–	Fish fed supplemented and non-supplemented LFM and HPS diets vs control high fish meal (HFM) diet (77 d)	Distal intestine	None of the supplements (tauro-cholate, bovine bile salt, taurine, lecithin or supplement mix) resolved gut inflammation or improved performance of fish	Kortner et al., 2016
Zebrafish (*Danio rerio*)	–	Supplementation of 50% soybean meal (SBM) with lactoferrin (LF, 0.5, 1 and 1.5 g/kg)	*Edwardsiella tarda*	Fish fed supplemented and non-supplemented SBM diet (4 d) were exposed to bacteria for 5 h, sampled 4 d post-infection	Intestine	High levels of LF inclusion reduced gut inflammation and increased survival of infected fish, indicating improved immunity	Ulloa et al., 2016

The early response at days 3 and 5 of dietary manipulation were dominated by immune-related transcripts, with gut function related genes being enriched from 5 days onwards. For immune genes, GTPase IMAP family members, regulators of T cell and B cell function and NF-kB-related genes were clearly increased in expression. The transcripts with reduced expression were related to functions including endocytosis, transport and metabolic processes, suggesting dysfunction of the intestinal epithelium and altered intestinal function.

Long-term feeding on high SBM diet was characterised by activation of T cell mediated processes via up-regulation of the CD86 antigen, cytotoxic T lymphocyte-associated protein 4, interleukin-18 (IL-18) and IL-22, while inflammation and respiratory burst were indicated by the increased expression of genes in the TNF- and NF-kB-mediated responses (De Santis et al., 2015). These responses could reflect the increased translocation of luminal bacteria, viruses and antigens across the intestinal epithelium, as indicated by increased expression of pathways for clathrin-mediated endocytosis signalling, macropinocytosis signalling and virus entry via endocytic pathways (Król et al., 2016).

The SBM is now commonly used as a model for inducing gut inflammation in fish intestine (e.g., Baeverfjord and Krogdahl, 1996, Krogdahl et al., 2003, Hu et al., 2016). For use in aquaculture feeds (especially for piscivourous fish), SBM is further purified to soy protein concentrate (SPC) by alcohol washing, which removes saponins, some of the major ANFs in raw soy material.

SPC-rich diets do not typically modify intestinal histology or enrich genes associated with SBM-induced enteritis (Tacchi et al., 2012, Król et al., 2016). The direct effect of soysaponins is complex as when fed to fish alone or with combination with corn gluten, sunflower, rapeseed or horsebean proteins - no or little inflammation was observed, whereas when soysaponins were used with pea protein, significant histological and transcriptional changes were observed (Kortner et al., 2012). These results suggest that the interactions between different AFNs are also important and the mechanisms by which different ANFs affect gut health are not fully understood. One possible explanation could be the increased intestinal permeability and response to various antigens associated with different types of plant materials (Knudsen et al., 2008, Penn et al., 2011, Chikwati et al., 2012, Krogdahl et al., 2015). Other plant protein concentrates used at high levels such as faba bean (bean protein concentrate, BPC) do not generally contain saponins. Instead, BPC is characterised by high levels of condensed tannins and the presence of faba bean-specific glucosides such as vicine and convicine. Importantly, both diets with high levels of SBM or BPC induced the gut inflammatory disease, but the transcriptomic analysis of the tissue indicated substantially different diet-specific responses, with a small core group of genes representing generic inflammation responses (Król et al., 2016). The shared response genes represent putative markers for inflammation across different inflammatory inducers.

#### 3.3.2 Vegetable oils

Development of effective strategies to replace fish oil with vegetable oils has been hindered by the difference in their fatty acid profiles, especially in the content of n-3 long-chain polyunsaturated fatty acids (n-3 LC-PUFAs) such as EPA and DHA, which are abundant in fish oil but not synthesised by terrestrial plants (Hixson, 2014). These fatty acids are essential or conditionally essential for all vertebrates and have well-established effects on immune and inflammatory processes in humans, including decreased production of pro-inflammatory cytokines and eicosanoids, enhanced phagocytosis and reduced leucocyte-endothelial adhesive interactions (Calder, 2013). These effects have been interpreted in the context of reducing inflammation that would enable inflamed tissues to return to homeostasis (Calder, 2006). In human nutrition, the most widely available dietary source of EPA and DHA is oily fish, such as salmon, herring, mackerel and sardines. The fish used as sources do not actually produce EPA or DHA, but instead accumulate them by consuming either marine phytoplankton (the primary source of n-3 LC-PUFAs), prey fish with EPA/DHA already accumulated or an aquaculture feed containing sufficient levels of fish oil as an ingredient. In contrast, vegetable oils shape the tissue fatty acid profile differently, generating fish that are typically low in EPA/DHA and high in n-6 LC-PUFAs, thus making them less desirable for human consumption (Gil et al., 2012). These low levels of dietary EPA/DHA may also have adverse effects on fish themselves, but the magnitude of these effects remains largely unknown. Research into the impacts of vegetable oils on fish health and immunity has recently gained momentum by combining feeding trials with large-scale analyses of gene expression performed on organs most likely affected by changes in the dietary fatty acid profile (Table 4).

**Table 4:** Fish studies using omics technologies to evaluate effects of vegetable oils on immunity.

Fish species	Omics technology	Dietary manipulation	Disease challenge	Comparison and sampling	Tissue analysed	Main findings	Reference
Atlantic salmon (*Salmo salar*)	Transcriptomics (microarray) and proteomics	Diet with 100% vegetable oil (VO, blend of rapeseed, palm and Camelina oils), with fish oil (FO) diet as control	–	Lean and fat fish fed VO vs FO diets (55 weeks)	Pyloric caeca	Both diet and genotype had limited effects on immune gene expression, with VO diet up-regulating transcript for liver-expressed antimicrobial peptide 2	Morais et al., 2012b
Atlantic salmon (*Salmo salar*)	Transcriptomics (microarray)	Diet with 100% vegetable oil (VO, blend of rapeseed, palm and Camelina oils), with fish oil (FO) diet as control	–	Lean and fat fish fed VO vs FO diets (55 weeks)	Liver	Diet had larger effects on immune gene expression than genotype, with majority of genes related to processes of both innate and adaptive immunity up-regulated in fish fed VO, apart from T cell and leukotriene B4 (LTB4) receptors that were down-regulated	Morais et al., 2011
Atlantic salmon (*Salmo salar*)	Transcriptomics (microarray)	Diet with 100% oil from Camelina (100COSEFM10CM), with 100% fish oil (FO) diet as control	–	Fish fed 100COSEFM10CM vs FO diets (16 weeks)	Liver	100COSEFM10CM diet induced significant changes in immune gene expression (e.g., up-regulation of CD200 and down-regulation of CD209 antigen-like protein E, lect-2 and chitinase 3), suggesting that fish fed Camelina oil diet were immuno-suppressed	Xue et al., 2015
Atlantic salmon (*Salmo salar*)	Transcriptomics (microarray)	2 diets with wild-type Camelina oil (WCO) and engineered EPA Camelina oil (ECO), with fish oil (FO) diet as control	–	Fish fed WCO vs ECO vs FO diets (7 weeks)	Pyloric caeca	Both WCO and ECO diets had similar but relatively small effects on immune gene expression, including up-regulation of T cell receptor and MHC II transcripts and down-regulation of neutrophil cytosolic factor 1	Betancor et al., 2015b
Atlantic salmon (*Salmo salar*)	Transcriptomics (microarray)	2 diets with wild-type Camelina oil (WCO) and engineered EPA Camelina oil (ECO), with fish oil (FO) diet as control	–	Fish fed WCO vs ECO vs FO diets (7 weeks)	Liver	Both WCO and ECO diets had similar but relatively small effects on expression of immune-related genes	Betancor et al., 2015a
Atlantic salmon (*Salmo salar*)	Transcriptomics (microarray)	2 diets with wild-type Camelina oil (WCO) and engineered EPA/DHA Camelina oil (DCO), with fish oil (FO) diet as control	–	Fish fed WCO vs DCO vs FO diets (11 weeks)	Liver	Both WCO and DCO diets had no effect on immune gene expression, suggesting no changes in fish immune status due to Camelina oils	Betancor et al., 2016
Atlantic cod (*Gadus morhua*)	Transcriptomics (microarray)	Diet with 66% oil from Camelina (C66), with 100% fish oil (FO) diet as control		Fish fed C66 vs FO diets (12 weeks)	Mid intestine	C66 diet induced subtle changes in expression of limited number of immune genes, including up-regulation of viperin, barrier-to-autointegration factor and interferon-induced protein 44	Morais et al., 2012a
Atlantic cod (*Gadus morhua*)	Transcriptomics (microarray)	2 diets with 40% and 80% oil from Camelina (40CO and 80CO), with 100% fish oil (FO) diet as control	Viral mimic polyriboinosinic polyribocytidylic acid (pIC)	Fish fed 40CO, 80CO and FO diets for 67 days were injected with pIC or PBS, sampled 0 and 24 h post-infection	Spleen	Both 40CO and 80 CO diets had no impact on immune gene expression before and after infection	Booman et al., 2014
Gilthead sea bream (*Sparus aurata*)	Transcriptomics (microarray)	Diet with vegetable oil (66VO, 66% fish oil replacement), with 100% fish oil (FO) diet as control	*Enteromyxum leei* (myxosporean parasite)	Fish fed 66VO and FO diets for 9 months were infected with parasite or kept unexposed, sampled 102 days post-infection	Distal intestine	Diet 66VO had no effects on transcriptome in unexposed fish, but substantially altered immune gene expression in infected fish, which correlated with increased progression of disease	Calduch-Giner et al., 2012

Most studies so far were conducted on Atlantic salmon (besides Atlantic cod and gilthead sea bream), using vegetable oil (either singly or as blends) from *Camelina sativa* (both wild-type and genetically modified) and focusing on either intestine (pyloric caeca and mid or distal guts) or liver, with one study targeting spleen. The replacement of fish oil with vegetable oils varied from 40 to 100% and the feeding trials lasted from 67 days to 55 weeks. Two of these studies investigated also the interaction between diet and genotype (fish selected for high and low lipid contents) (Morais et al., 2011, Morais et al., 2012b), while all other experiments were performed on non-selected fish. On two occasions, the feeding trails were followed by the disease challenge, using either viral mimic polyriboinosinic polyribocytidylic acid (pIC) (Booman et al., 2014) or *Enteromyxum leei* (myxosporean parasite) (Calduch-Giner et al., 2012).

The tissues for the transcriptomic evaluation of the effects of vegetable oils on fish health were selected based on their contribution to digestion, metabolism and immune system, including gut as the main site of the direct exposure to nutrients and foreign antigenic material, liver as the main metabolic organ and spleen as the important lymphoid organ. Although none of the studies listed in Table 4 compared the expression of immune genes between different tissues, the majority of changes induced by the exposure of non-challenged fish to wild-type vegetable oils were described in liver rather than in gut or spleen. These findings are consistent with the involvement of the liver in detoxification, modulation of immune responses, as well as the production of inflammatory mediators (Knolle and Gerken, 2000). Importantly, the replacement of 66% of fish oil with vegetable oil in the diet of gilthead sea bream had no effects on the gut transcriptome in unchallenged fish, but substantially altered the expression of immune genes in infected fish (Calduch-Giner et al., 2012), highlighting the need for testing the novel diets in the context of exposure to pathogens.

## 4 Future perspectives

Although the interplay between nutrition and immune system is well recognised, basic and applied research on the interactions between diet and health in fish is lagging behind the mammalian studies. To fully understand the repercussions of aquaculture feeds on fish physiology, a shift in approach is required to determine the molecular and cellular pathways that regulate responses to different diets. The new omics technologies, especially transcriptomics coupled with full genome sequences, offer enormous potential to investigate the complex relationship between fish nutrition and immunity, both in health and disease. However even though the field is advancing rapidly, there are a number of major gaps in the knowledge that need to be addressed. One of the major challenges is for example the relationship between the nutritional content of aquaculture feeds, fish intestinal microbiota and the resultant metabolites, and how these metabolites modified differently by different diets impact fish heath and their resistance to pathogens. Fish microbiota studies are advancing with deep sequencing approaches, but as yet there is little interpretation of the findings of such studies in the context of fish immune status and health. Understanding the mechanisms that underpin the links between diet, intestinal microbiota and fish health will almost certainly become a major focus in the next few years.

Early life development and the dietary impacts on development of the intestinal and immune system are still far from clear. Such research is important as it may lead to designing strategies for nutritional programming and intestinal tolerance at the time of first feeding. The relationship between diet and ontogeny of the immune system will also require the knowledge of both trans-generational epigenetic control of immune gene expression as well as life-long epigenetic control of immune genes expression established during the time of first feeding. Such experiments could expand the use of zebrafish as a model species and also exploit the lines of transgenic fish, which allows the development and homing of specific immune cell types to be examined. Other models include intestinal cell culture, either primary or stable cell cultures, that will help to explore the direct impacts of dietary components (or microbial metabolites) on intestinal immune capacity.

Many studies described in this review provide strong evidence for the interplay between nutrition and immunity at the level of innate immune components. However, little is known about the impacts of dietary manipulations on the adaptive immune responses, such as activation of T and B lymphocytes. The adaptive immune responses are central to long-term effects of diet on fish health and resistance to pathogens. Furthermore, understanding the link between nutrition and adaptive immune system is essential for developing strategies for oral vaccination and the improvement of antigen uptake and memory.

The final future perspective is how these omics technologies can be integrated with the ambition of generating predictive models for diet, immune system and health outcomes. Such work requires improved genome annotation, the knowledge of immune cell type-specific responses and mathematical computational expertise, which can then be combined and used to dissect the molecular mechanisms underlying the diet-immunity interactions, leading to improved health of farmed fish and sustainable aquaculture.
